# Microstructure and Oxidation Behavior of Metal V Films Deposited by Magnetron Sputtering

**DOI:** 10.3390/ma12030425

**Published:** 2019-01-30

**Authors:** Song Zhang, Tingting Wang, Ziyu Zhang, Jun Li, Rong Tu, Qiang Shen, Chuanbin Wang, Guoqiang Luo, Lianmeng Zhang

**Affiliations:** 1 State Key Laboratory of Advanced Technology for Materials Synthesis and Processing, Wuhan University of Technology, 122 Luoshi Road, Wuhan 430070, China; superkobe0104@gmail.com (S.Z.); 18202767268@163.com (T.W.); zhangziyu1108@foxmail.com (Z.Z.); sqqf@263.net (Q.S.); wangcb@whut.edu.cn (C.W.); luoguoqiang1980@sina.com (G.L.); LMZhang@whut.edu.cn (L.Z.); 2 National Key Laboratory for Shock Wave and Detonation Physics, Institute of Fluid Physics, P.O. Box 919-102, Mianyang 621900, China; lijun102@caep.cn

**Keywords:** Vanadium films, magnetron sputtering, substrate temperature (*T*_s_), target–substrate distance (*D*_t–s_), microstructure, oxidation behavior

## Abstract

Direct-current magnetron sputtering (DCMS) was applied to prepare vanadium (V) films on Si substrate. The influence of substrate temperature (*T*_s_) and target–substrate distance (*D*_t–s_) on phase structure and surface morphology of V films were investigated by X-ray diffraction (XRD), scanning electron microscopy (SEM), atomic force microscope (AFM) and transmission electron microscopy (TEM). The results show that the crystallinity of the V films increases with increasing *T*_s_ and decreasing *D*_t–s_. The film deposited at *T*_s_ = 400 °C and *D*_t–s_ = 60 mm exhibits the best crystallinity and <111> preferred orientation with a regular tetrahedral surface morphology. Oxidation behavior of the V thin films has also been studied by X-ray photoelectron spectroscopy (XPS).

## 1. Introduction

Vanadium (V), a strategic rare metal material, has several excellent physical and chemical properties, when compared with other metals; these include high hardness, high melting point, good thermal and electrical conductivity, and good corrosion resistance. Initially, it was used as an alloying element to increase the strength of vanadium high-carbon steel alloys [1]. In recent years, metallic V thin films have attracted more attention and have been used on objects to achieve isolation from the surrounding environment, considering their advantages of high temperature resistance, hydrochloric acid, and sulfuric resistance [2]. In addition, hybrid nanocomposites are a rapidly growing field of science in pursuit of novel materials with tailored functionality and improved properties [3]. It might be possible to fabricate thin film nano-patterns for optical, electrical or sensing applications using this material [3,4]. There has been extensive research on bulk vanadium [5,6,7,8]. In contrast, very little work has been reported on V thin films, whose properties may differ from those of bulk because of the small size in the direction of thickness and the interrupted continuity by the presence of surfaces and interfaces. Li et al. [9] deposited V films on aluminum alloy substrates using high power pulse magnetron sputtering (HPPMS) and found that the *D*_t-s_ has a critical effect on surface morphology, microstructure, deposition rate, and corrosion resistance. Wei et al. [10] successfully produced fully dense, nano-structured V thin films using a two-step consolidation method. They concluded that the grain size of nano-structured V thin films is in the order of 100 nm, identified by X-ray diffraction and transmission electron microscopy. They measured the mechanical properties of nano-structured V thin films consolidated at 600 °C using both quasi-static and dynamic compression tests and reported that the Vickers hardness is 6.0 GPa [10]. Vanadium is harder than most metals, but oxidizes even in ambient air. Mamun et al. [1] fabricated V thin films with different thicknesses on Si substrates by magnetron sputtering, and investigated the natural oxidation behavior and mechanical properties of the deposits. It was reported that by measuring the 30% depth of the film thickness and eliminating the influence of the substrate on the film properties, the hardness of oxidized films depicted less variation than un-oxidized films. This result shows that oxidation of the V film may affect its mechanical properties. Previous researchers have focused more on the mechanical properties and corrosion resistance of V films. However, few reports have systematically studied the structure and morphology of V films and the relationship between structure and oxidation behavior. 

Methods such as evaporation, molecular beam epitaxy, sputtering, and pulsed laser deposition have been used to prepare V films [11,12,13,14]. Among them, the sputtering method is widely used because of its good adhesion between the film and substrate, controllable film thickness, high repeatability, and high purity of the prepared film. 

In this work, the DC magnetron sputtering (DCMS) technique was adopted to prepare the V films. The effects of substrate temperature (*T*_s_) and target-substrate distance (*D*_t-s_) on microstructure and surface morphology of the V films were investigated by various testing methods. We then discussed the relationship between structure and natural oxidation behavior.

## 2. Materials and Methods 

The V films were deposited by DC magnetron sputtering on Si (100). After 20-min ultra-sonic bath in acetone, the Si substrate (10 × 10 × 0.5 mm^3^) was fixed on the substrate holder. As the sputtering source material, a V-metal target (purity: 99.9%) with a diameter of 2 inches was used. The vacuum chamber was evacuated to a base pressure lower than 9.0 × 10^−4^ Pa. Argon gas was introduced into the chamber to maintain a pressure of 0.5 Pa. The sputtering power was fixed at 100 W. Before starting the deposition, the V target was pre-sputtered for 5 mins to remove contamination from the target surface. The substrate temperature (*T*_s_) varied from 25 °C to 400 °C. The target–substrate distance (*D*_t–s_) changed from 60 mm to 100 mm. The sputtering time was 60 mins.

Structural properties of the films were analyzed using X-ray diffraction with a multipurpose platform attachment (XRD, Ultima III, Rigaku, Tokyo, Japan) with Cu*Kα* source at 40 kV and 40 mA. Microstructural observation of the deposited films was carried out using transmission electron microscopy (TEM, JEM-3010, JEOL Ltd., Tokyo, Japan) at an accelerating voltage of 200 kV. The chemical state of elements in the films was analyzed using X-ray photoelectron spectroscopy (XPS, ESCALAB 250Xi, Thermo Fischer, Waltham, MA, USA). Surface morphology of the films were studied using field emission scanning electron microscopy (FESEM, Quanta-250, FEI, Houston, TX, USA) at an accelerating voltage of 25 kV and atomic force microscope (AFM, Multimode 8, Bruker, Karlsruhe, Germany).

## 3. Results

**Figure 1:**
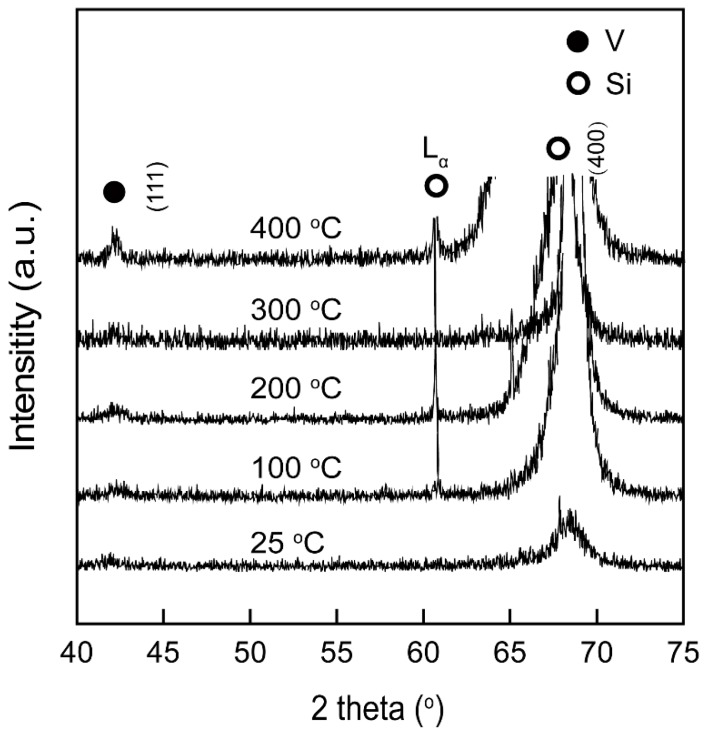
XRD patterns of V films deposited on Si (100) at *D*_t-s_ = 60 mm with different *T*_s_.

Figure 1 shows the XRD patterns of V films deposited on Si (100) substrate at *D*_t-s_ = 60 mm with different *T*_s_. At *T*_s_ of 25 and 100 °C, the phase of as-deposited films is amorphous, whereas at *T*_s_ of 200 °C, only V (111) and Si (100) peaks could be observed, indicating that the deposited V film is totally <111>-orientation, which means that the preferred is <111>-orientation. The relative intensity of the V (111) films increases on increasing *T*_s_ from 200 °C to 400 °C. The results indicate that high substrate temperatures result in better crystallinity of the films. It is generally understood that the deposited atoms on the substrate have more opportunity to move around on the substrate, forming larger crystallites in the film, when a higher *T*_s_ is applied [15].

**Figure 2:**
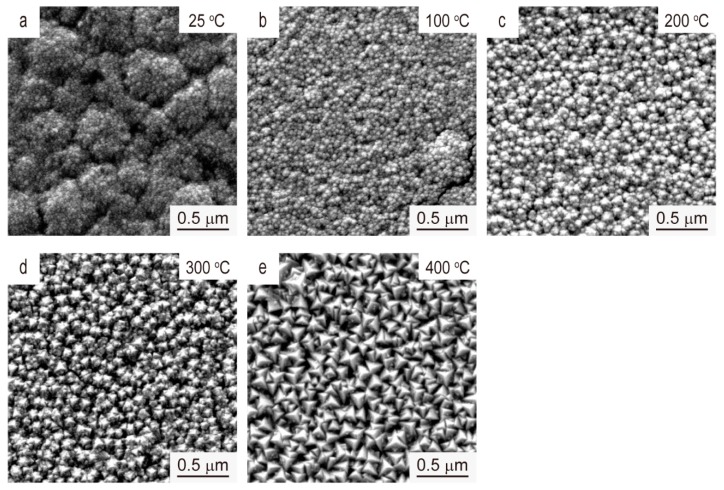
SEM images of the V films deposited on Si (100) at *D*_t-s_ = 60 mm, *T*_s_ = (**a**) 25 °C, (**b**) 100 °C, (**c**) 200 °C, (**d**) 300 °C, and (**e**) 400 °C.

The SEM images in Figure 2 represents the surface morphologies of V films deposited on Si (100) substrate at *D*_t-s_ = 60 mm with different *T*_s_. The surface of the V film prepared at 25 °C exhibited features similar to a cauliflower. As *T*_s_ increased to 100 °C, the surface of the V films gradually flattens and particle size of V films gradually increases. With further increase of *T*_s_, the surface morphologies of V films evolves from a sphere in Figure 2a,b to a polyhedron in Figure 2c,d, and then to a triangular pyramid in Figure 2e. It is worth mentioning that the unique triangular pyramid-like morphology of the V film represents a preferred <111>-orientation [16,17,18], corresponding to the XRD results in Figure 1.

**Figure 3:**
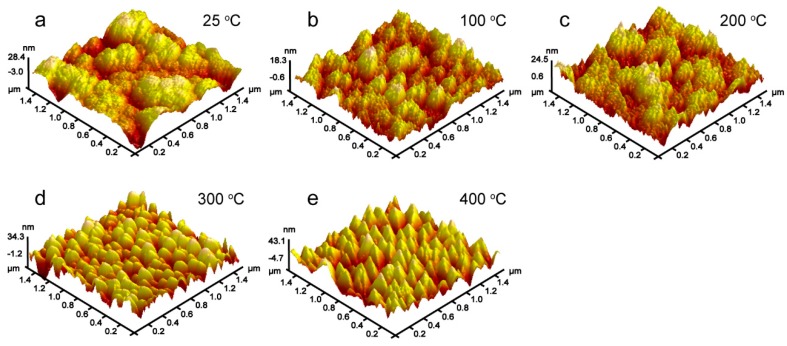
AFM results of V films deposited on Si (100) at *D*_t-s_ = 60 mm, *T*_s_ = (**a**) 25 °C, (**b**) 100 °C, (**c**) 200 °C, (**d**) 300 °C, and (**e**) 400 °C.

**Figure 4:**
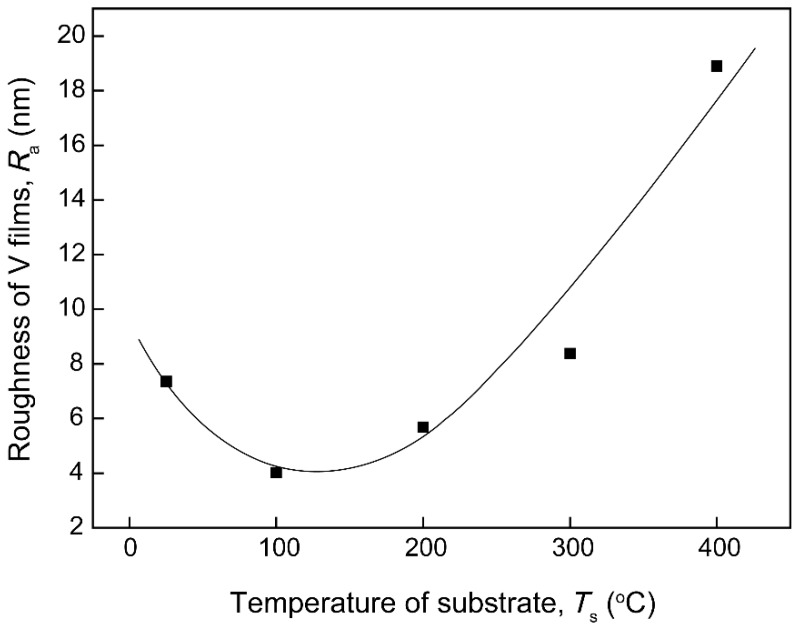
Effects of substrate temperatures (*T*_s_) on the roughness (*R*_a_) of V films on Si (100).

Figure 3 shows atomic force micrograph images of the V films deposited at *D*_t-s_ = 60 mm with different *T*_s_. It was found that the surface particles of the films gradually became larger and the uniformity of the films gradually increased, which was consistent with the SEM results. In addition, the effect of substrate temperatures (*T*_s_) on the mean surface roughness (*R*_a_) of the V films was shown in Figure 4. It was found that *R*_a_ decreased first and then increased on increasing *T*_s_. The decrease in *R*_a_ is caused by the fact that high *T*_s_ promotes the arrival of V atoms in the desired location due to enhanced diffusion movement, resulting in a smoother surface when *T*_s_ is below 100 °C [19]. However, when *T*_s_ exceeds 100 °C, the increased particle size of the surface leads to an increase in the roughness of the V films [20].

**Figure 5:**
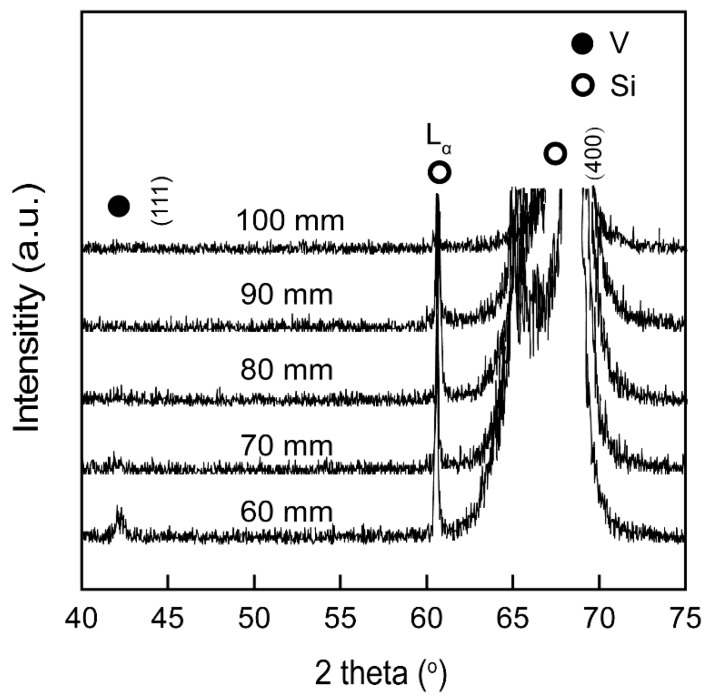
XRD patterns of V films deposited on Si (100) at *T*_s_ = 400 °C with different *D*_t-s_.

The XRD patterns of V films deposited at *T*_s_ = 400 °C with different *D*_t-s_ were shown in Figure 5. The relative intensities of V (111) peaks decreased with increasing the *D*_t-s_, indicating that the crystallinity of the film was deteriorated. The average free path of the particles in the vacuum chamber is constant at a certain working pressure and sputtering power. When the substrate is kept closer to the target, the particles have higher energy since they undergo less number of collisions. Hence, the particles are more mobile on the substrate surface, leading to good crystallinity of the film. When *D*_t-s_ is increased, the sputtered particles undergo more collisions and hence will have a lower mobility and this results in less crystalline films [21].

**Figure 6:**
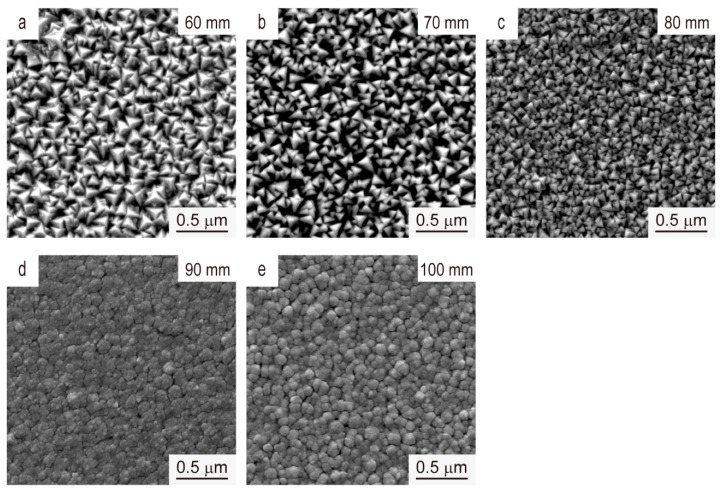
SEM images of the V films deposited on Si (100) at *T*_s_ = 400 °C, *D*_t-s_ = (**a**) 60 mm, (**b**) 70 mm, (**c**) 80 mm, (**d**) 90 mm, and (**e**) 100 mm.

Figure 6 represents the surface morphologies of V films deposited on the Si (100) substrate at *T*_s_ = 400 °C with different *D*_t-s_. The particle size of the V films decreases on increasing *D*_t-s_. The variation of surface morphologies is related to the reduced crystallinity and preferred orientation of the V films, in consistent with the XRD data in Figure 5.

**Figure 7:**
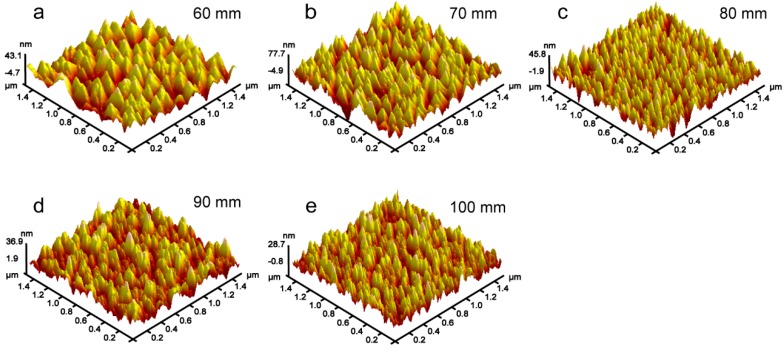
AFM results of V films deposited on Si (100) at *T*_s_ = 400 °C, *D*_t-s_ = (**a**) 60 mm, (**b**) 70 mm, (**c**) 80 mm, (**d**) 90 mm, and (**e**) 100 mm.

**Figure 8:**
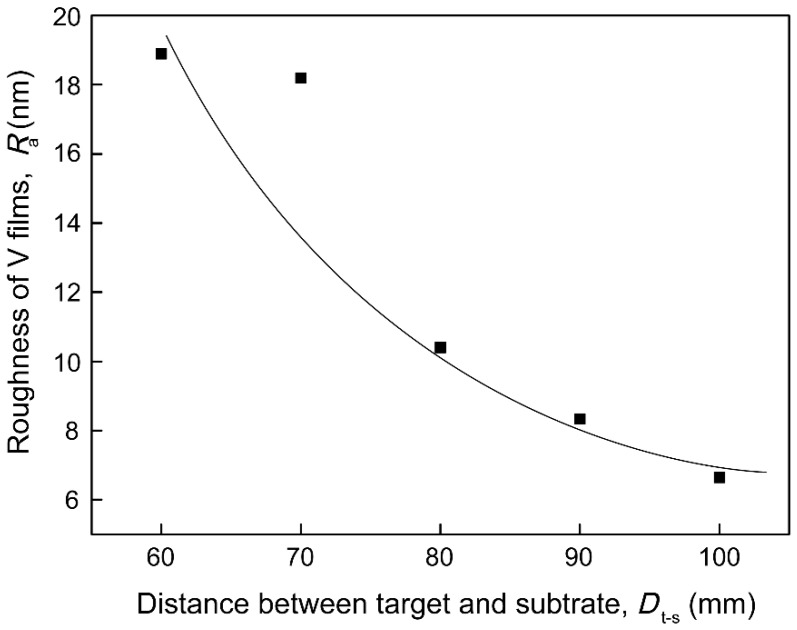
Effects of target-substrate distances (*D*_t-s_) on the roughness (*R*_a_) of V films on Si (100).

The AFM images of V films deposited on Si (100) substrate at *T*_s_ = 400 °C with different *D*_t-s_ are shown in Figure 7. The size of the triangular pyramid-shaped particles gradually becomes smaller as *D*_t-s_ increases. The particles on the surface of the V film prepared above 80 mm gradually transform into a cone shape. The effects of target-substrate distances (*D*_t-s_) on the roughness (*R*_a_) of V films deposited on Si (100) substrate were investigated in Figure 8. We can draw a conclusion that the *R*_a_ of V films decreases with an increase in *D*_t-s_. The decreased *R*_a_ may be attributed to the decreased particle size caused by the weakened preferred orientation and competitive growth of grains.

**Figure 9:**
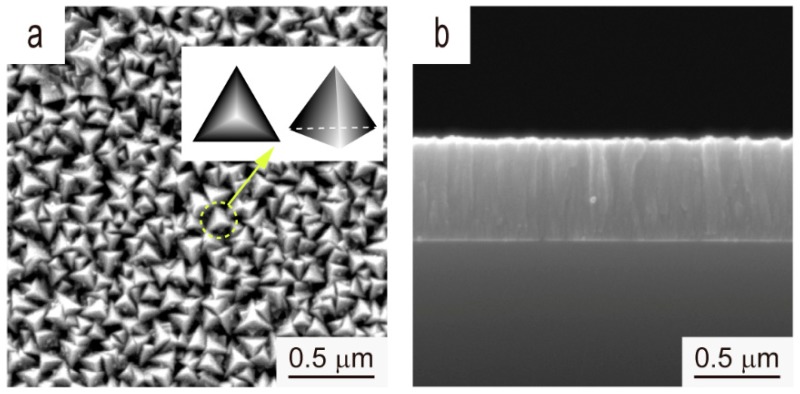
(**a**) Surface and (**b**) cross-sectional SEM images of the V film deposited on Si (100) at *T*_s_ = 400 °C and *D*_t-s_ = 60 mm.

Figure 9 shows the surface and cross-sectional SEM images of the V film on Si (100) deposited at *T*_s_ = 400 °C and *D*_t-s_ = 60 mm. The morphology of the as-fabricated V film is a triangular pyramid containing the slowest-growing {111} planes, and with the fastest growing <111>-direction [22]. The cross-section of an as-fabricated V film shows that the film exhibits a columnar grain structure. 

**Figure 10:**
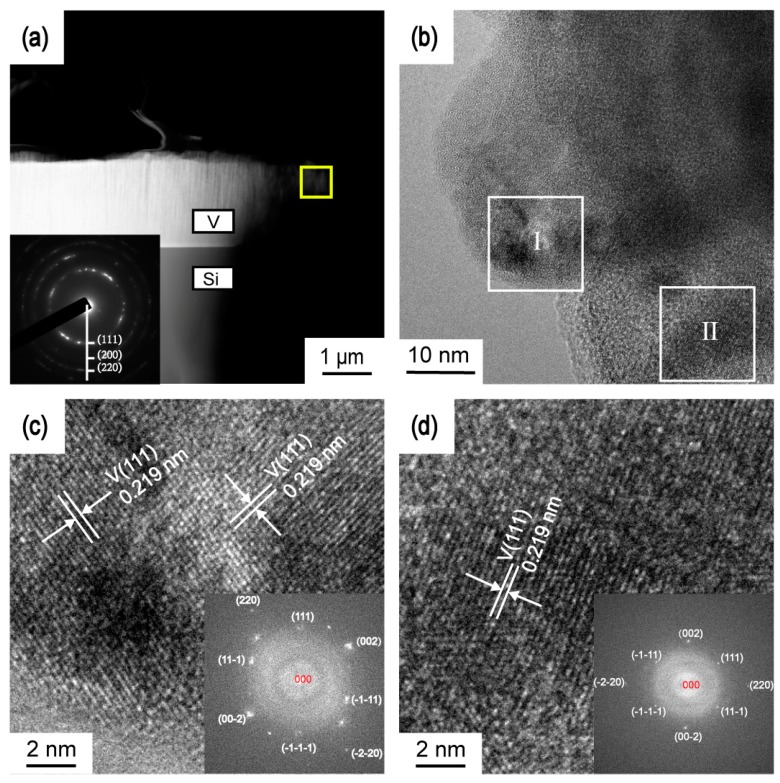
(**a**) Cross-sectional TEM image of the V film deposited on Si (100) at *T*_s_ = 400 °C and *D*_t-s_ = 60 mm and SAED pattern of the yellow square area in insert, (**b**) TEM image of the yellow square area in (**a**), (**c**) HRTEM image and Fast Fourier Transform (FFT) of region I, (**d**) HRTEM image and Fast Fourier Transform (FFT) of region II.

Transmission electron microscopy (TEM) was used to further investigate the structure of the V film on Si (100) deposited at *T*_s_ = 400 °C and *D*_t-s_ = 60 mm in Figure 10. Figure 10a illustrates the cross-sectional TEM images of the V film deposited at *T*_s_ = 400 °C and *D*_t-s_ = 60 mm. The cross section exhibits a columnar structure with a flat top. The selective area electron diffraction (SAED) pattern of the yellow square area inserted in Figure 10a demonstrated the presence of metallic V nanoparticles in the V films. The texture of V was a face-centered cubic (FCC) structure, indexed as (111), (200) and (220). The TEM image in Figure 10b shows that the V film exhibits single crystal features in a small range. Figure 10c,d are HRTEM images of region I and II, respectively. The lattice fringe spacing of 0.219 nm is related to (111) plane of metallic vanadium. The insets in Figure 10c,d were a Fast Fourier Transform (FFT) of the corresponding HRTEM images, which reveals the (slightly tilted) hexagonal symmetry of the lattice of metallic vanadium.

**Figure 11:**
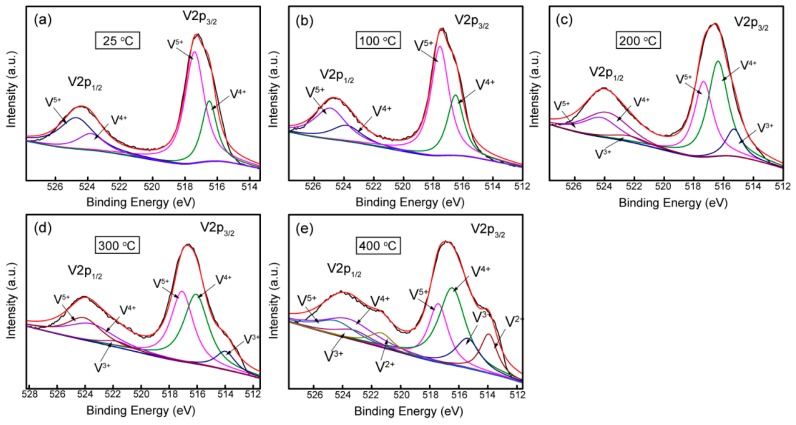
XPS spectra of V films deposited on Si (100) at *D*_t-s_ = 60 mm, *T*_s_ = (**a**) 25 °C, (**b**) 100 °C, (**c**) 200 °C, (**d**) 300 °C, and (**e**) 400 °C.

**Figure 12:**
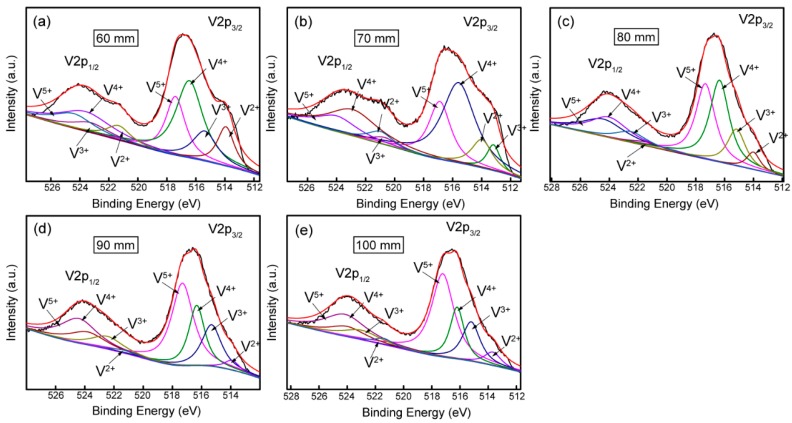
XPS spectra of V films deposited on Si (100) at *T*_s_ = 400 °C, *D*_t-s_ = (**a**) 60 mm, (**b**) 70 mm, (**c**) 80 mm, (**d**) 90 mm, and (**e**) 100 mm.

**Figure 13:**
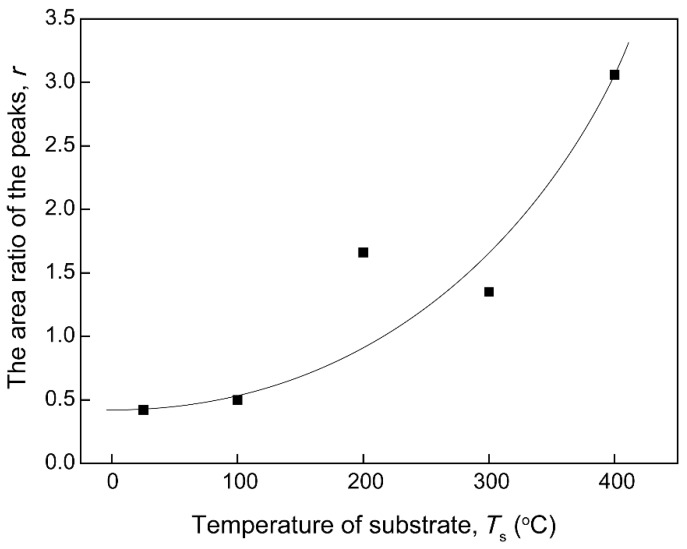
Effects of substrate temperatures (*T*_s_) on the area ratio (*r*) of the peaks of V films on Si (100).

**Figure 14:**
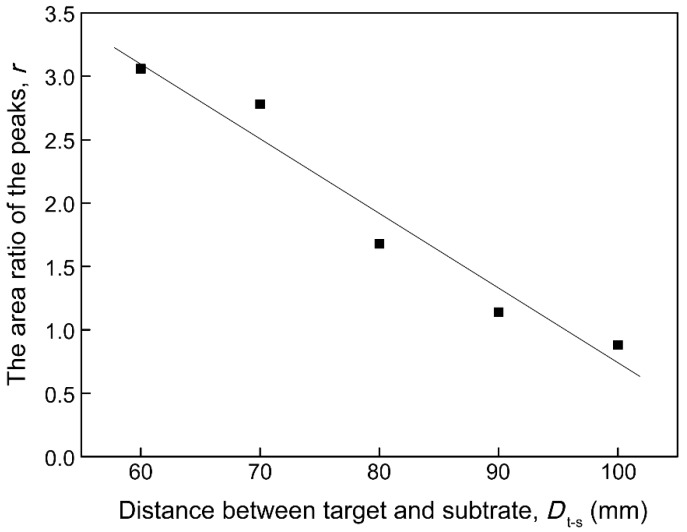
Effects of target-substrate distances (*D*_t-s_) on the area ratio (*r*) of the peaks of V films on Si (100).

XPS analysis was performed to study the composition of natural oxide layer of the V films placed in air for 1 month. XPS spectra of V 2p of the V films deposited at different parameters shown in Figure 11 and Figure 12 indicates the existence of multivalent vanadium ions (V^2+^, V^3+^, V^4+^, V^5+^). XPS fitting results for the V 2p_3/2_ peaks of vanadium oxides reported in this study referred to a form listed by E. Hryha [23]. It could be seen roughly that the XPS results confirm the presence of a natural oxidation layer of V films placed in air for 1 month, and the oxide layers grown on different V films have different compositions. The composition of natural oxidation layers can reflect the oxidation resistance of the V films. During the oxidation of vanadium, the O^2−^ needs to grasp most electrons from metallic V to form vanadium oxides with the highest valance, requiring more energy compared with other valences. In other words, the less V^5+^ appeared in the oxide layers, the stronger the oxidation resistance of V films in the surrounding atmosphere. In this paper, we classified V^2+^, V^3+^, and V^4+^ as low-valence particles and V^5+^ as high-valence particles. On the XPS spectrum, the number of each kind of particle can be represented by the area of its corresponding peak. The area ratio (*r*) of the peaks of low valence V^2+^, V^3+^, and V^4+^ to the highest valence V^5+^ [ *r* = (SV^2+^ + SV^3+^ + SV^4+^)/SV^5+^ ] (SV^2+^, SV^3+^, SV^4+^, and SV^5+^ represent the areas of the peaks of V^2+^, V^3+^, V^4+^, and V^5+^, respectively.) were calculated to indicate the oxidation resistance of the V films prepared at different parameters in Figure 13 and Figure 14. The larger the value of *r*, the stronger the oxidation resistance of the film.

Figure 11 shows the composition of the oxide layers of V films prepared at *D*_t-s_ = 60 mm with different *T*_s_. At *T*_s_ of 25 and 100 °C, the XPS spectra shows the presence of V^4+^ and V^5+^ valances. When *T*_s_ increases to 200 °C and 300 °C, V^3+^ appears on the surface of V films. At *T*_s_ of 400 °C, there are not only V^3+^, V^4+^, V^5+^ valances, but also V^2+^ in the oxide layer. We found that an obvious shift of V2p_2/3_ peaks toward the low binding energy was induced by increasing *T*_s_. As can be seen from the curve in Figure 12, the area ratio (*r*) of the peaks increases with an increase in *T*_s_. This result indicates that the oxidation resistance of V film gradually becomes stronger as *T*_s_ increases. This increased oxidation resistance of V films may be related to the smaller specific surface area of the large particles on the film surface at higher *T*_s_ (Figure 2). In addition, the oxidation resistance of V films also should be due to the good crystallinity of films with larger grains obtained at a high *T*_s_ [24], confirmed by the results of XRD (Figure 1). The diffusion of oxygen is weakened due to an increase in grain size and a decrease in interfacial area as the crystallinity increases.

XPS spectra of V 2p and the area ratio (*r*) of the peaks of V films deposited at *T*_s_ = 400 °C with different *D*_t-s_ are shown in Figure 13 and Figure 14, respectively. It can be seen from Figure 13 and Figure 14 that the relative quantity of V^5+^ in the highest valence state increases as *D*_t-s_ increases, and V^3+^ and V^2+^ in the low valence state gradually decreases. This result indicates that as *D*_t-s_ increases, the oxidation resistance of the V film gradually weakens. The oxidation resistance should be due to the reduced crystallinity and preferred orientation of V films with smaller grains obtained at larger *D*_t-s_.

## 4. Conclusions

Structure and surface morphology have been studied on V films grown on Si substrates with various *T*_s_ and *D*_t-s_. We obtained films with good crystallinity and preferred <111>-orientation at higher *T*_s_ and lower *D*_t-s_. The shape of the surface particles gradually become more regular and the size of the particles decreased with increasing *T*_s_ and decreasing *D*_t-s_. The morphology of the V films prepared at *T*_s_ = 400 °C and *D*_t-s_ = 60 mm presents a triangular pyramid shape, with typical characteristics of preferred <111>-orientation. In addition, we found that films with better crystallinity and larger surface particles are less susceptible to oxidation in air.
